# A modular microfluidic bioreactor to investigate plant cell–cell interactions

**DOI:** 10.1007/s00709-021-01650-0

**Published:** 2021-05-02

**Authors:** T. Finkbeiner, C. Manz, M. L. Raorane, C. Metzger, L. Schmidt-Speicher, N. Shen, R. Ahrens, J. Maisch, P. Nick, A. E. Guber

**Affiliations:** 1 Institute of Microstructure Technology, Karlsruhe Institute of Technology, Hermann-von-Helmholtz-Platz 1, 76344 Eggenstein-Leopoldshafen, Germany; 2 Molecular Cell Biology, Botanical Institute, Karlsruhe Institute of Technology, Fritz-Haber-Weg 4, 76131 Karlsruhe, Germany; 3 Present Address: Institute of Pharmacy, Martin-Luther-University Halle-Wittenberg, Biosynthesis of active substances, Hoher Weg 8, 06120 Halle (Saale), Germany

**Keywords:** *Catharanthus roseus*, Cell communication, Microfluidic bioreactor for plant cells, *Nicotiana tabacum* L. BY-2, Plant cell fermentation (PCF)

## Abstract

Plants produce a wide variety of secondary metabolites, which often are of interest to pharmaceutical and nutraceutical industry. Plant-cell cultures allow producing these metabolites in a standardised manner, independently from various biotic and abiotic factors difficult to control during conventional cultivation. However, plant-cell fermentation proves to be very difficult, since these chemically complex compounds often result from the interaction of different biosynthetic pathways operating in different cell types. To simulate such interactions in cultured cells is a challenge. Here, we present a microfluidic bioreactor for plant-cell cultivation to mimic the cell–cell interactions occurring in real plant tissues. In a modular set-up of several microfluidic bioreactors, different cell types can connect through a flow that transports signals or metabolites from module to module. The fabrication of the chip includes hot embossing of a polycarbonate housing and subsequent integration of a porous membrane and in-plane tube fittings in a two-step ultrasonic welding process. The resulting microfluidic chip is biocompatible and transparent. Simulation of mass transfer for the nutrient sucrose predicts a sufficient nutrient supply through the membrane. We demonstrate the potential of this chip for plant cell biology in three proof-of-concept applications. First, we use the chip to show that tobacco BY-2 cells in suspension divide depending on a “quorum-sensing factor” secreted by proliferating cells. Second, we show that a combination of two *Catharanthus roseus* cell strains with complementary metabolic potency allows obtaining vindoline, a precursor of the anti-tumour compound vincristine. Third, we extend the approach to operationalise secretion of phytotoxins by the fungus *Neofusicoccum parvum* as a step towards systems to screen for interorganismal chemical signalling.

## Introduction

In multicellular organisms, division, growth and differentiation of individual cells depend on intercellular communication. Cells can communicate either by direct contact or by means of extracellular signalling. Establishment of such specific cell-to-cell interactions is pivotal to the development of an organism, including metabolic differentiation. This is particularly relevant for secondary metabolism, which, by definition, comprises those metabolic activities that are not essential for the cell itself but relevant for the organism as an entity (Kössel 1891). The regulation of secondary metabolism, thus, requires mechanisms to gear the metabolism of specific cells towards producing complex secondary metabolic products.

Secondary metabolites are natural products produced by an organism to play a specific role but not essential for its growth, development and reproduction. Plants produce over 200,000 unique chemical compounds, which help them in defence as well as interspecies competition (Pyne et al. 2019). Many of these compounds are pharmacologically active and thus very valuable. However, these secondary metabolites are available in minute concentration *in planta* (Miettinen et al. 2014; Vidensek et al. 1990). Often, these valuable compounds are difficult to produce by chemical synthesis, because they are structurally and stereochemically complex. Therefore, extraction and processing from their natural sources is still the main approach to obtain these compounds. As a result, the attempt to exploit sufficient quantities of these scarcely occurring molecules from the natural resources can lead to overexploitation of these plants, in some cases pushing them to the verge of extinction (Lin et al. 2018). Alternative strategies to produce these compounds are mandatory, therefore. Although total chemical synthesis or semi-synthetic approaches have been successful, such strategies are frequently far from being or economically feasible (Lin et al. 2018; Thornburg et al 2017). In some cases, metabolic engineering based on transgenic approaches can increase the accumulation of these compounds in vivo but has to meet its own challenges such as negative impact of the altered metabolism on growth and development of the plant (for reviews, see Wilson and Roberts 2014; Tatsis and O'Connor 2016). Alternatively, heterologous hosts allow obtaining the desired secondary products (Tsuruta et al. 2009; Westfall et al. 2012). However, especially for smaller quantities, plant-based production systems are easier to handle and, therefore, more cost-efficient compared to mammalian, yeast, or bacterial expression systems (Daniell et al. 2001). For instance, to produce the anti-Malaria compound artemisinin in plants turned out to be cheaper than heterologous systems (Peplow 2016). Biotechnological production of secondary compounds in plant cell cultures (so-called plant cell fermentation) would, thus, provide an attractive alternative and is already used for diverse products, because it enables scale-up from experimental set-ups to industrial scale production (Ochoa-Villarreal et al. 2016; Tekoah et al. 2015). The best-known example for large-scale technological production of a plant compound is the production of the anti-tumour compound Paclitaxel from cell cultures of the yew *Taxus chinensis* (Malik et al. 2011).

**Fig. 1:**
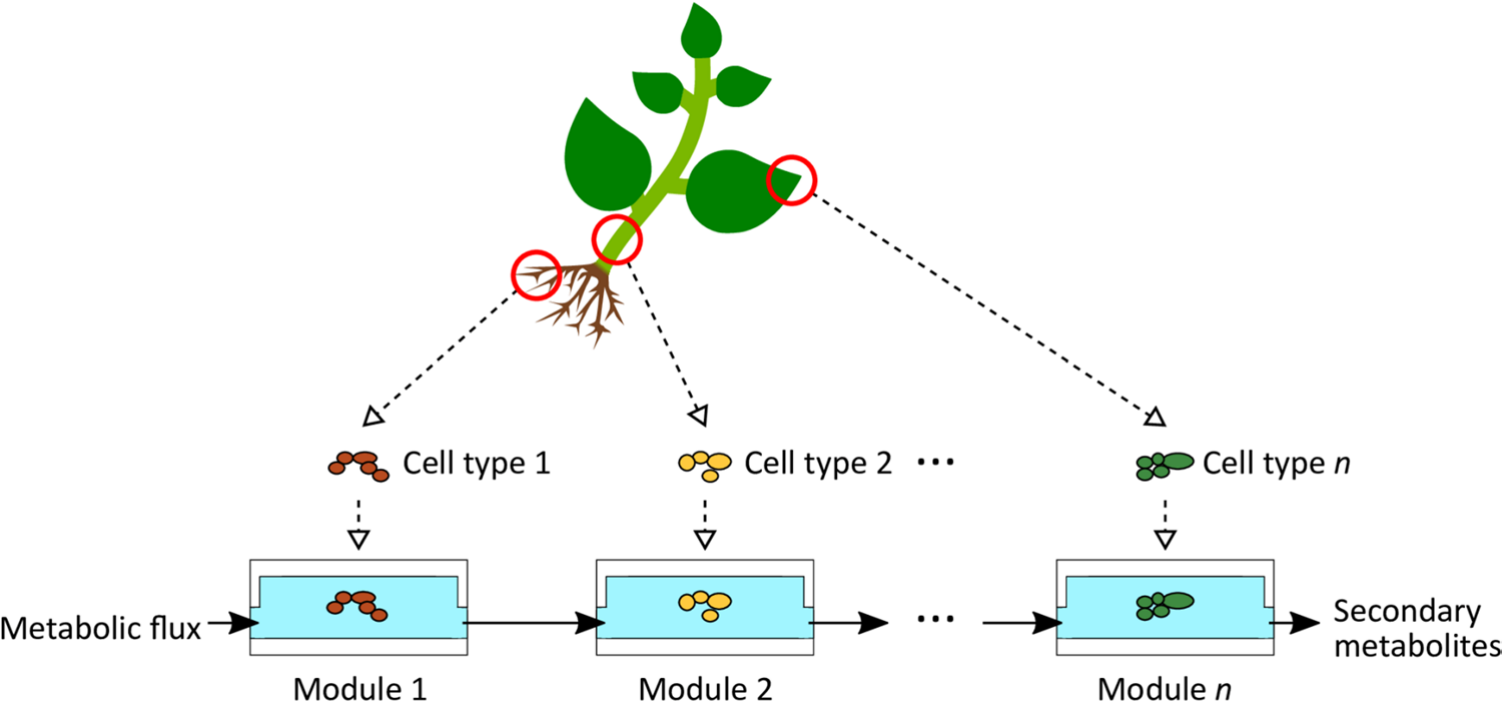
Conceptual framework of the study. To simulate metabolic interactions in a real plant tissue, we couple different cell types by a microfluidic flow

Despite its prospects, plant cell fermentation also has to face its specific challenges. One limitation for the biotechnological exploitation of plant cell cultures is the compartmentalization of secondary metabolism to different cell types (Ziegler and Facchini 2008). Conventionally, biotechnological production follows the principle one bioreactor—one product. However, this fails to mimic the intercellular compartmentalisation in the natural situation requiring the interaction between different cell types to obtain the final product. For instance, the accumulation of the anti-Alzheimer compound nornicotine in tobacco BY-2 cells became possible only, when two different cell strains overexpressing key enzymes of the pathway were allowed to communicate (Rajabi et al. 2017, 2020). Likewise, the vinca alkaloids produced by *Catharanthus roseus* require interaction between four different cell types to culminate in the accumulation of these anti-cancer compounds (Simkin et al. 2013). Thus, it is not astonishing that the synthesis of these precious compounds in cell cultures of *Catharanthus roseus* has remained for almost half a century without significant success (Rischer et al. 2006). In the plant tissue, accumulation of value-giving plant secondary compounds often requires the coupling of cell types with different metabolic activity by a dynamic flow. To mimic this coupling in cell suspensions is far from trivial. Technological solutions for this drawback in plant cell fermentation might derive from microfluidics technology. This technology allows coupling of different cell types with specific metabolic potencies by a metabolic flow, thereby simulating situation in a real plant tissue (Fig. 1). Flexible combination of such modules could yield complex as well as novel metabolites. Microfluidic systems have allowed to integrate metabolic microcompartments in bottom-up systemic biology systems (Beneyton et al. 2018), but have meanwhile also entered plant cell biology, e.g. to study pollen-tube growth (Nezhad et al. 2014), or to follow expression of fluorescent reporters in roots of *Arabidopsis* seedlings (Busch et al. 2012). In this context, we have developed a microfluidic chip that allows cultivation of walled plant cells. For the model system tobacco BY-2, we could show that the cells in this system maintained their physiological status over an entire period and even recapitulated all details of their normal developmental cycle (Maisch et al. 2016).

In the current paper, we now extent this microfluidic chamber system for cell–cell interactions. We fabricate the system, made of biocompatible and transparent thermoplastics through ultrasonic welding providing a fast, efficient and biocompatible joining technique (Ehrenstein and Ahlers-Hestermann 2004; Grewell et al. 2003; Potente 2004). This system is endowed with a user-friendly and tight connector type (Finkbeiner et al. 2019), such that it allows for convenient combination of different modules harbouring different cell populations through a common metabolic or signalling flow. We present three proof-of-concept applications for the use of this microfluidic chip. As first example, we study a factor secreted by densely growing tobacco BY-2 cells that can initiate proliferation of non-proliferating recipient cells. Second, we use the chip system to combine two cell strains of *Catharanthus roseus* differing by their metabolic potency, and to generate vindoline, a rate-limiting precursor of the value-giving vincristine. In the third case, we mimic a minimal “ecosystem on chip” by coupling the endophytic fungus *Neofusicoccum parvum* with tobacco BY-2 cells to operationalise secreted phytotoxins.

## Materials and methods

### Design of the chip

**Fig. 2:**
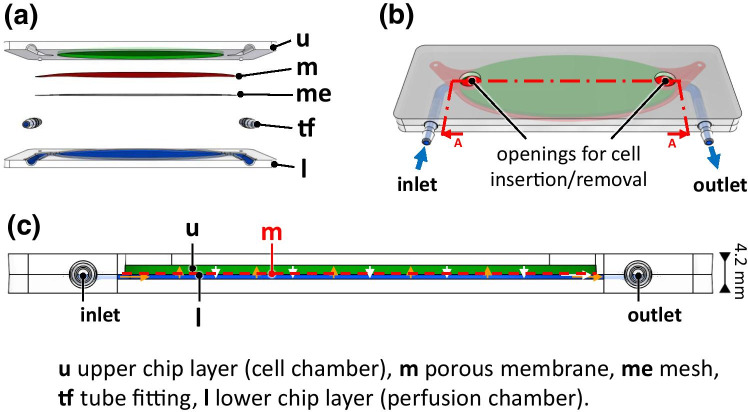
Chip design. **a** Explosion view with upper and lower chip layer, membrane, mesh and tube fittings. **b** Perspective view, blue arrows indicate the flow direction of the nutrient solution. **c** Cross section of section A-A from (**b**), orange arrows indicate the direction of mass transfer of the nutrients whereas white arrows indicate the direction of mass transfer of products

The chip consisted of two layers (Fig. 2a). The lower layer was a perfusion chamber for the nutrient solution. The upper layer consisted of a cell chamber that contained plant cells. A porous membrane separated both layers (Fig. 2c). This allowed keeping the cells in place and enabling nutrient supply and mass exchange by the flow of nutrient solution. This set-up enabled the modular combination of several chips with a common metabolic perfusion flow. In principle, plant cells of the first cell chamber can secrete metabolites to the perfusion flow, which reach the plant cells in the next modules. Thus, signals and metabolites can migrate from module to module. Insertion and removal of cell was possible through two openings with a diameter of 5 mm in the cell chamber, whereas the perfusion chamber had one inlet and one outlet to perfuse with nutrient solution (Fig. 2b).

The modular and user-friendly set-up of the chips was possible through tube fittings N210-9 (Value Plastics dba Nordson MEDICAL, USA) with an inner diameter of 1.6 mm inner diameter as fluidic connectors as described elsewhere (Finkbeiner et al. 2019).

The dimensions of the chip corresponded to a standard microscope slide (25 mm × 75 mm), and a cell-chamber height of 1 mm. These dimensions provided short diffusion paths of the nutrients or products but sufficient space for the cells that are around 40–50 µm in height, at an average length of 250 µm (Kreppenhofer 2013; Maisch and Nick 2007). The volume was set to 800 µl offering direct comparison with experiments in 48-well titre plates. To avoid adhesion of air bubbles in corners of the cell chamber, the design was elliptical with a large area to facilitate the simultaneous observation of many cells. The total height of the chip was 4.2 mm to provide sufficient working distance for microscopic observation.

To achieve the combination of high transparency, biocompatibility, and hardness, we used the thermoplastic polymer polycarbonate (PC) Makrolon® GP clear 099 (Bayer Material Science AG, Germany) as material for the chip housing (Domininghaus 2012). The membrane had to be transparent and made up of pores small enough to retain the cells in the cell chamber. Additionally, it had to be thin as well and sufficiently porous to sustain short diffusion paths for nutrients and products. Therefore, we chose a polyethylene terephthalate (PET) membrane PET5020030 (Sterlitech Corporation, USA) with a thickness of 19 µm and a pore diameter of 5.0 µm.

### Computational fluid dynamics

To validate the suitability of the membrane, we modelled the mass transfer of sucrose as nutrient from the perfusion chamber through the porous membrane into the cell chamber using a simulation software (COMSOL Multiphysics®, COMSOL Inc., Sweden). We simulated the membrane as porous filter (Chung et al. 2014) rather than as a porous medium (Loskill et al. 2017; Vereshchagina et al. 2013). Due to the long computation times, we restricted the simulation to 2D using the module ‘transport of diluted species’. Furthermore, we assumed a ‘laminar flow’ model with a Reynolds number of 2.37 × 10^−7^ deriving from a medium flow velocity of 2.84 × 10^−4^ m s^−1^, a channel height of 0.50 mm, a canal width of 2.35 mm, and a flow rate of 20 $\upmu \mathrm{l}{\cdot \min}^{-1}$ .

The geometry was integrated into the model (COMSOL Multiphysics®) which allowed calculating the pore distance *L*_*p–p*_ according to Chung et al. (2014) as

1 $${L}_{p-p}=\sqrt{\frac{{\pi r}^{2}}{\mathrm{sin}\left(60\right)\cdot \varepsilon }}$$

with a membrane porosity *ε* that depends on the number of pores *n*_Pores_. We could determine pore number by counting a membrane area of 230 μm × 143 µm (= 3.29 × 10^−2^ mm^2^) using SEM micrographs arriving at an average of 116.7 pores was received, leading to a pore distance of *L*_*p–p*_ = 18.09 µm under the consideration of vertical cylindrical pores compared to *L*_*p–p*_ = 16.99 µm specified by the manufacturer.

To calculate the diffusion coefficient of sucrose in water, we used the Stokes–Einstein equation:

2 $${D}_{\mathrm{Sucrose}}=\frac{{k}_{\mathrm{B}}{T}_{0}}{6\pi {\eta }_{\mathrm{Sucrose},\mathrm{ Water}}{R}_{\mathrm{Sucrose}}}$$

with a value of 5.5 × 10^−10^ m as molecular radius of sucrose (Kashima and Imai 2017). The viscosity *η*_Sucrose, Water_ of a 30 g l^−1^ sucrose-water solution was determined in triple measurements with a rheometer (RheoStress 300, ThermoHaake®, Germany) with a conical titanium plate (60 mm diameter, 1°, 52 μm gap) leading to a value for *η*_Sucrose, Water_ of 1.0277 mPa s. Since the Boltzmann constant is *k*_B_ = 1.38 × 10^−23^ $\mathrm{J}\cdot{\mathrm{K}^{-1}}$, we get for room temperature (*T*_0_ = 294.15 K) an estimated diffusion coefficient *D*_Sucrose_ = 3.81 × 10^−10^ m^2^ s^−1^, which is in accordance with the published record (Kashima and Imai 2017; Ueadaira and Uedaira 1969). Finally, we calculated the concentration maps using a fine two-dimensional grid by means of the backward differentiation formula solver at time intervals of 0.5 s over a total calculation interval of 250 s.

### Fabrication of the chip parts

We fabricated the chip from semi-finished polycarbonate plates of 3 mm thickness by hot embossing with a brass moulding tool (i-sys Mikro- und Feinwerktechnik GmbH, Germany) for fast and reliable replication of the chip parts (Maisch et al. 2016; Worgull 2009). To facilitate the demoulding process, we adjusted a demoulding angle of 3° at all vertical edges in the brass tool and used a polished chrome counter plate to ensure transparency of the chip parts. The membrane and the woven fabric were prepared using a laser cutter VLS3.50 (Universal Laser Systems, USA).

### Assembly technology

We assembled the microfluidic bioreactor by ultrasonic welding (PS DIALOG digital control, Herrmann Ultraschalltechnik GmbH & Co. KG, Germany) in a two-step process. This procedure allows integrating the tube fittings and the membrane in plane, reducing total chip height to 4.2 mm.

**Fig. 3:**
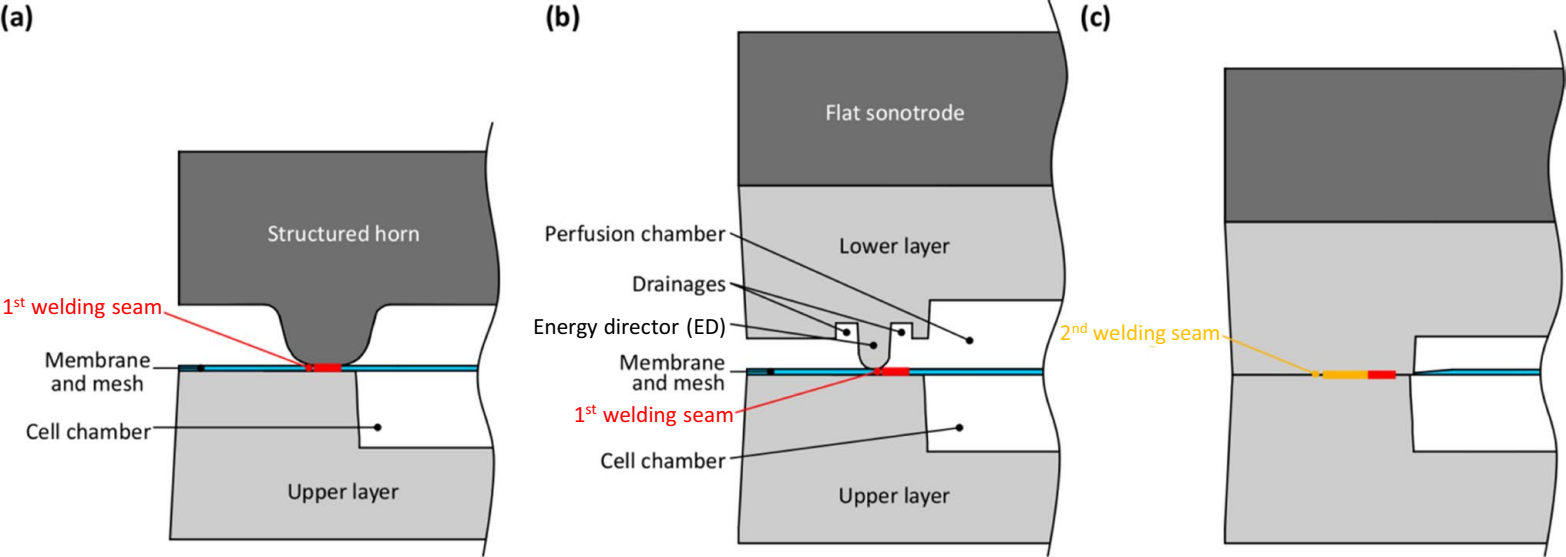
Two-step process for ultrasonic welding of the chip. **a** First step to weld the membrane and the mesh with a structured horn on the upper layer with cell chamber. **b**, **c** Second step to weld the lower layer with perfusion chamber and the tube fittings

As first step, we welded the membrane directly on the chip housing with the cell chamber by means of the woven PET fabric (Fig. 3a). We generated a defined first welding seam, using a structured titanium horn (Herrmann Ultraschalltechnik GmbH & Co. KG, Germany) of 400 µm in height with a rounded energy director (ED), and a self-transformation of 1:1.7, in combination with a 1:1.5 booster. Due to the circular cross section of the fabric fibres, the point contacts of the fabric on the polymer membrane acted like additional miniaturised EDs. This procedure enables ultrasonic welding of very thin polymer sheets, because weldability decreases with decreasing thickness (Potente 2004; Rotheiser 2009). Furthermore, it allows for more homogeneous and gentle welding of the membrane. To avoid damage from fast oscillations of the thin layer, we used a comparatively low force of 200 N and a total amplitude of 13.3 µm to weld membrane and mesh (Grewell et al. 2003).

In the second step, we welded the perfusion chamber onto the membrane-sealed cell chamber (Fig. 3b, c), which sealed the whole system to the outside. In the same step, we integrated the tube fittings as described elsewhere (Finkbeiner et al. 2019). To avoid further tension on the membrane, the second welding seam was placed with an offset of 0.28 mm and an overlap of 0.07 mm in relation to the first welding seam. Height and width of the ED were 400 µm with a 200-µm radius edge rounding and was surrounded by drainages on both sides for excess melt. For this step, we used a flat horn (Herrmann Ultraschalltechnik GmbH & Co. KG, Germany) with a self-transformation of 1:1.5 in combination with a 1:2.5 booster resulting in a total amplitude of 21.9 µm. The clamping force was 500 N to compensate the slope due to the tube fittings, and the welding force was larger than in the first step (300 N). We verified the welding seam of the first welding step by SEM (SEM Supra 60VP, Zeiss, Germany) and, in addition, analysed membrane planarity with a digital stereo microscope (KEYENCE VHX-6000, KEYENCE Corporation, USA).

### Quality control of the fabricated chip

To examine the tightness of the chips, we connected the tube fittings of the perfusion chamber to compressed nitrogen, while the cell chamber openings were closed using 3 M Polyester Film Tape 851 (3 M Deutschland GmbH, Germany). Subsequently, we immersed the whole chip in a beaker with water and tested for tightness as described elsewhere (Finkbeiner et al. 2019). In addition, we recorded pressure over flow rates of individual chips, or of two chips connected in series to validate a proper and leakage-free operation. For this purpose, we connected the chips to a syringe pump PHD ULTRA™ (Harvard Apparatus, USA) with a syringe containing water, steadily increasing flow rate to 30 $\mathrm{ml}{\cdot \min}^{-1}$  and visually checking for potential leakage of the chips.

### Use of the microfluidic chip to detect quorum sensing in BY-2

As a proof of concept for an interaction between two physiologically different cell populations, we used quorum sensing in BY-2. In suspension, the cells stop proliferating at high dilutions but resume proliferation in response to cell-free medium from proliferating cells growing at sufficient density. To simulate this phenomenon technically, we coupled two chips into a microfluidic series, using stationary cells at day 7 after subcultivation. Two serially coupled chips were combined with cells in different dilutions (1:300 or 1:30 of a stationary culture) in different combinations (Fig. 6): Either donor chamber and recipient chamber at 1:300, both at 1:30, or the donor chamber at 1:30 and recipient chamber at 1:300. The set-up was circular, with a reservoir flask, feeding the two serial chips with MS medium and collecting the liquid again, after it had passed through the chip. The total volume was 30 ml, such that in steady state, the effective solution of the cells in the chamber would be multiplied by a factor of 30 ml (total volume) by 2 × 0.8 ml (the loaded volume in the two chambers). In the 1:30 set-up, this would correspond to a 560-fold dilution of a stationary culture, in the 1:300 set-up, accordingly 5600-fold. The cell cultures were cultivated using a modified Murashige-Skoog (Duchefa Biochemie, Netherlands) medium for 3 days, such that the cells could enter the proliferation phase (Huang et al. 2017). Four days later, at the end of the culture cycle, we removed the cells from the chip and determined their density in a haemocytometer (Paul Marienfeld GmbH & Co. KG, Germany). Data represent mean and standard error from three independent experimental series, with 5 technical replications per experiment. If the dense donor culture would produce the quorum-sensing factor, this should be manifested as a re-initiated proliferation of the diluted recipient cells. We connected the two chips by Tygon® tubing with an inner diameter of 1.6 mm (Reichelt Chemietechnik GmbH & Co., Germany) and perfused with culture medium at a flow rate of 20 $\upmu \mathrm{l}{\cdot \min}^{-1}$  in a circular flow (to facilitate accumulation of the quorum-sensing factor) driven by a 4-channel Ismatec REGLO Digital peristaltic pump (Cole-Parmer GmbH, Germany).

### Use of the microfluidic chip to generate metabolic synergy in *Catharanthus roseus*

We cultivated suspension cell strains of *Catharanthus roseus* (L.) G. Don (C1 and C4) originating from seeds of *Catharanthus roseus* plants in fresh and autoclaved growth medium containing Gamborg B5 salts (3.21 g l^−1^), sucrose (30 g l^−1^) and 2,4-D (5 mM), adjusted to pH 5.6. We subcultured cells weekly, by inoculating 3 g (fresh weight) of filtered cells into the growth medium (50 ml) in 250 ml polycarbonate Erlenmeyer flasks with filter caps (Corning GmbH, Kaiserslautern, Germany). The cells were incubated at 26 °C in the dark on a gyratory platform shaker (Heidolph Instruments GmbH, Germany) at 120 rpm. A volume of 800 µl suspension for each strain was loaded to a chip and combined in a circular flow at a flow rate of 30 $\upmu \mathrm{l}{\cdot \min}^{-1}$  as described above after elicitation with 100 MeJA (Duchefa Biochemie (Haarlem, Netherlands). After 2 weeks, the supernatant was frozen at − 20 °C and lyophilised for 3 days. After dissolving the lyophilisate with 1 ml of MetOH and ultrasonication for 2 min (amplitude 100%, 0.5 s pulse) using a high-efficiency ultrasound device (UP 100H, Hielscher Ultrasonics GmbH, Teltow, Germany). We removed all particulate matter by spinning the samples down for 10 min with 10,000 × *g* at 25 °C and filtering the supernatant through a 0.45-μm needle-type Chromafil PET-20/15 MS filter (Macherey–Nagel GmbH & Co. KG, Düren, Germany) into the autosampler vials (WIC4200, WICOM Germany GmbH, Heppenheim, Germany). Individual stock solutions of the alkaloid standards such as catharanthine, tabersonine, vindoline, vinblastine and vincristine were prepared at a concentration of 1 mg/ml in MetOH. These stock solutions and the alkaloid extracts were stored at − 20 °C for further analysis. For sensitive qualitative analysis by liquid chromatography-mass spectrometry (HPLC–DAD-ESI–MS/MS) of vinca alkaloids, we used a LXQ Linear Ion Trap MSn system (Thermo Fisher Scientific, Waltham, MA, USA) equipped with a Finnigan Surveyor HPLC–PDA. The extracts were separated on a Phenomenex Luna C18 column (4.6 mm × 250 mm, 5 μM particle size) with a gradient of 10 mM ammonium acetate, pH 6.0 (solvent A) and LC-grade MetOH (solvent B) as mobile phase using a flow rate of 500 $\upmu \mathrm{l}{\cdot \min}^{-1}$ . The eluent profile (% of solvent A/% of solvent B) was 0–5 min using a linear gradient from 30:70 to 10:90 and 5–23 min with an elution gradient from 10:90 to 30:70. We detected masses using an ion trap mass spectrometer coupled with electrospray ionisation, operating in a positive mode at a spray voltage of 4 kV, a capillary voltage of 33 V, a capillary temperature of 350 °C and a tube-lens voltage to 70 V. The full mass scan covered the range from *m*/*z* 100 to 1000.

### Use of the microfluidic chip to detect secreted fungal phytotoxins

To test whether the chip allows detecting the interaction between cells from different species through soluble factors, we combined tobacco BY-2 cells and the wood-decaying fungus *Neofusicoccum parvum*. To create a suspension culture of this fungus and to stimulate the release of phytotoxins, we transferred a plug of fungal mycelia (growing on potato dextrose agar) into liquid MS medium and maintained the suspension at biweekly intervals. The two life forms were growing in two separate microfluidic chips, connected with Tygon S3™ tubing (Saint-Gobain Performance Plastics, France). For perfusion, we used the standard BY-2 cultivation medium (Huang et al. 2017) at a flow rate of 30 $\upmu \mathrm{l}{\cdot \min}^{-1}$  driven by a 4-channel Ismatec REGLO Digital peristaltic pump (Cole-Parmer GmbH, Germany). Here, we used a unidirectional flow from a source flask through the chips into a waste receptacle. The cell chamber of the first chip contained 800 µl of a *N. parvum* suspension culture, the cell chamber of the second chip 800 µl of tobacco BY-2 cells. We used the *N. parvum* cells at day 1 post-subcultivation, the tobacco BY-2 cells at the day of subcultivation. After filling the cell chambers, we closed their openings with biocompatible tape (3 M Polyester Film Tape 851, 3 M Deutschland GmbH, Germany) and incubated the set-up for two additional days. To evaluate the experiment, we extracted the BY-2 cells from the cell chamber and quantified viability by the fluorescence diacetate assay (Widholm 1972). Briefly, we added 1% of a 50-mg ml^−1^ stock (in acetone) FDA dye and viewed directly. Living cells are fluorescent green, since cytoplasmic esterases cleave the non-fluorescent FDA into the fluorescent product fluorescein. Dead cells lacking this enzyme activity emit no fluorescence. The FDA signal was examined by an AxioImager Z.1 microscope (Zeiss, Jena, Germany), using the filter set 38 HE (excitation: 470 nm, beamsplitter: 495 nm and emission: 525 nm, Zeiss). To detect potential mortality due to the chip itself, we scored viability in a negative control where tobacco BY-2 cells were perfused omitting flow through fungal cells. Data represent three biological replicates counting 550 to 4000 cells per replicate using the analyse particle routine of the ImageJ software (NIH, USA, ver.1.53c, https://imagej.nih.gov/ij/).

## Results

### Characterisation of ultrasonic welding and fluidic properties

**Fig. 4:**
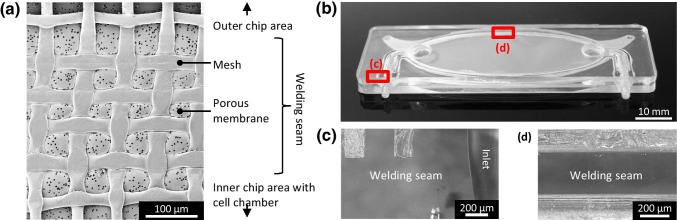
Welding seams. **a** Welding seam between membrane and mesh with the upper chip part housing the cell chamber after the first welding step. **b** Chip after the second welding step with the positions of welding seam between tube fitting and chip housing **(c)**, welding seam between the chip parts **(d)**

We assessed the homogeneity of the welding seam between mesh, membrane and the chip layer (Fig. 4a) and found that the mesh structure was fully welded through the membrane on the chip layer which validated the effect of the miniaturised energy director (ED) on the mesh structure. By ultrasonically embossing of the welding area at the chip layer by the structured horn prior to welding, we achieved a better planarity. Still, the fact that membrane planarity fluctuated by around 800 µm in average, indicated that the process of ultrasonic welding of such a thin membrane causes wrinkles.

This is due to the welding front that pulls and pushes the membrane heterogeneously. The second welding step culminated in the ready chip, which was 4.2 mm in height (Fig. 4b). This welding step produced transparent and tight welding seams between tube fittings and the two chip housings at the inlet of the perfusion chamber (Fig. 4c), as well as between the chip housings at the cell and perfusion chamber (Fig. 4d). After this step, the chip was ready to be autoclaved for the biological experiments. When the ED causes complete plasticity of membrane and mesh, a transparent welding seam results, and the chip remains tight up to 0.65 bar. Incomplete penetrance of the ED into membrane and mesh leads to leakage at much lower pressure (0.08 bar). We attempted to obtain even higher tightness than to 0.65 bar by using higher welding forces up to 500 N. However, this decreased optical quality and frequently resulted in membrane damage. Thus, each chip had to be tested individually for maximum pressure tightness. However, this drawback was not relevant for our applications. When we tested leakage over flow rate, we found that a flow rate of 20 $\upmu \mathrm{l}{\cdot \min}^{-1}$ , which was sufficient for our experiments, did not lead to any leakage. To reach the threshold pressure of 0.08 bar we had to increase flow rate to about 21 $\mathrm{ml}{\cdot \min}^{-1}$ , i.e. to three magnitudes higher than the flow rate used for the experiments.

### Simulation of diffusion

**Fig. 5:**
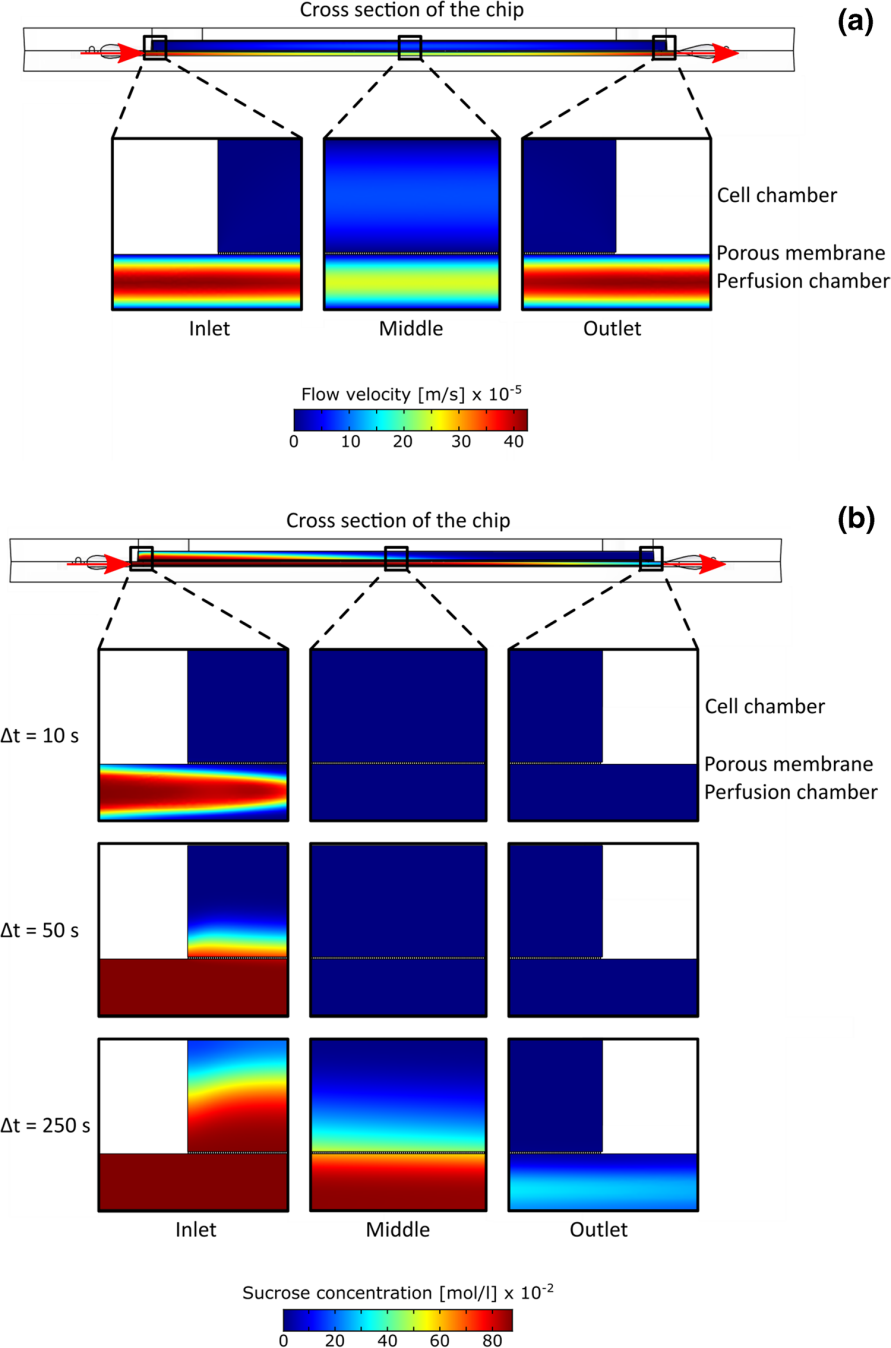
Modelling of flow and diffusion in the chip. **a** Simulation of flow velocity. **b** Predicted concentration profiles of sucrose in water after 0 s; 50 s and 250 s

We simulated the performance of the chip assuming a laminar flow profile (Fig. 5a), which predicted a decrease of velocity with increasing cross-sectional area of perfusion chamber and cell chamber. Despite the membrane barrier, a flow should occur into the cell chamber. The flow velocity in the perfusion chamber should decrease in the middle of the chip, whereas the flow velocity should increase in the cell chamber to a maximum of 1.97 × 10^−5^ m s^−1^ in the middle of the cell chamber. This flow velocity would not cause any significant shear stress to the cells. We simulated the distribution of sucrose in 2D profile for both, perfusion and cell chamber, after 10 s, 50 s, and 250 s (Fig. 5b). This simulation predicts a slow diffusion of sucrose through the pores of the membrane. Due to the diffusion of sucrose molecules over the membrane into the cell chamber, a convection force should result, which will push the molecules from the end of the cell chamber back to the perfusion chamber. This force would contribute to the flow rate in the cell chamber mentioned above, and accelerate the diffusion process. Since a peristaltic pump induces regular fluctuations of speed in the flow of the medium, oscillating shear-forces might alter cellular behaviour. While this holds true even more in conventional suspension cultures, where cells move on an orbital shaker, we have minimised shear forces by administering only 20 µl min^−1^. Since we did not observe perturbations even of subtle aspects of physiology (Maisch et al. 2016) under substantially higher flow rates (> 70 µl min^−1^), we think that the biological impact of shear forces at this low flow rate are negligible.

### Use of the chip to detect secreted proliferation factors in tobacco BY-2

**Fig. 6:**
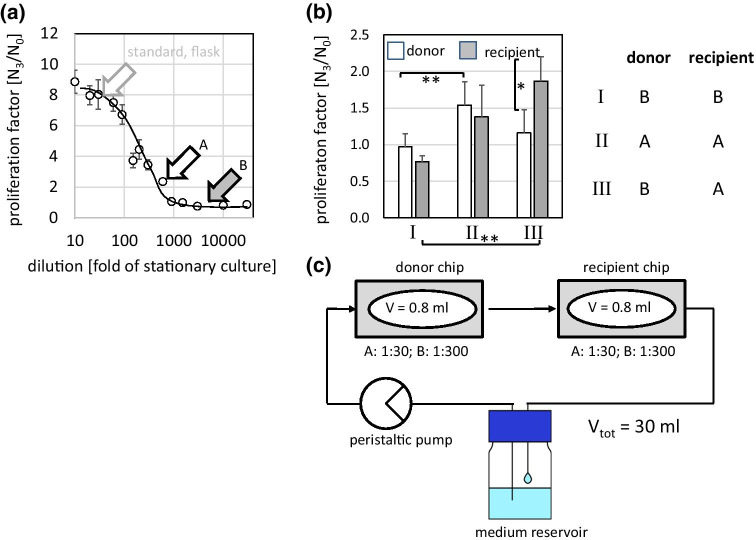
Demonstration of quorum sensing in tobacco BY-2 cells. **a** Dose–response relation for the proliferation factor (defined as ratio of cell density at day 3 over cell density in the initial inoculum for cells grown in suspension). The arrows indicate the densities prevailing in the chip experiment for the two loading schemes A and B shown in (**c**). **b** Proliferation factors in donor and recipient chip for different loading schemes. *P < 0.05 and **P < 0.01—significant levels. **c** Schematic representation of the experimental set-up. Data represent mean and standard error from three independent experimental series, with 5 technical replications per experiment

Suspension cells exchange soluble signals that regulate development and proliferation. While individual cells can survive and proliferate on solid medium, they halt proliferation in suspension, if the density drops under a certain threshold. To map this response for tobacco BY-2 cells, we first determined the proliferation factor (scoring the ratio between the cell densities at day 3 over that of the initial inoculum) in dependence on the dilution of the inoculum (Fig. 6a). For the standard protocol, this dilution would be 1:30 leading to a proliferation factor of around 8. This factor dropped rapidly, if the dilution exceeded 1:100 dropping to 1:1 (corresponding to the absence of proliferation) for a dilution of 1:1000. For a dilution of around 1:600 (corresponding to the steady state in a circular set up, when both chambers are filled with a standard inoculum of 1:30 of the stationary culture), this proliferation factor dropped already to around 2, and for the tenfold diluted set-up, cells did not proliferate. In the next step, we followed proliferation in two chips in a circular set-up (Fig. 6c). Here, we tested three combinations. In configuration I, we filled both chips with a tenfold higher dilution of the suspension (1:300 of a stationary culture, in steady state of the set-up corresponding to a ~ 5600-fold dilution). In configuration II, both chips contained a standard suspension (1:30 of a stationary culture, in steady state of the set-up corresponding to a ~ 560-fold dilution). In configuration III, which was the actual probing set-up, the donor chamber harboured a standard (1:30) suspension, while the recipient chamber contained a tenfold diluted (1:300) suspension. In fact, the outcome was significantly different between these three set-ups (Fig. 6b). At the higher dilution of 1:300 in both chambers, the proliferation was absent, when both chambers harboured the standard dilution, proliferation was around 50%, which is comparable to the value in a flask culture (Fig. 6a, arrow A). When the donor chamber at (1:30) fed a recipient chamber at (1:300), the proliferation shifted unexpectedly—the factor in the donor chamber was lower as compared to the set-up with both chambers in standard dilution, albeit not significantly. However, the proliferation in the diluted (1:300) recipient chamber increased significantly to almost twofold. This is in stark contrast to set-up I, where the same dilution did not yield any proliferation. Thus, when the donor chamber contains cells in a higher concentration, this will stimulate the proliferation in the otherwise silent cells of the recipient chamber.

### Use of the chip to detect metabolic synergy of different *Catharanthus* cell strains

**Fig. 7:**
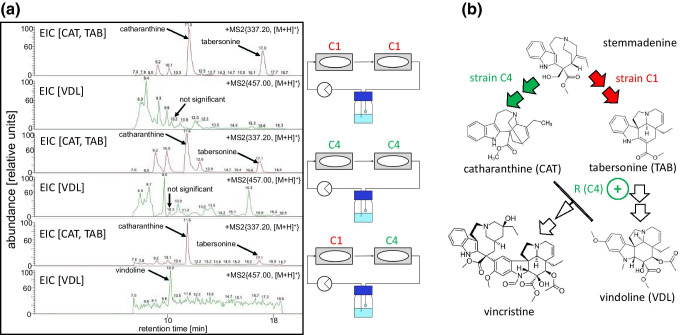
Demonstration of metabolic synergy in two cell strains of *Catharanthus roseus* differing in their metabolic potencies. **a** Representative HPLC–DAD-ESI–MS/MS spectra for intracellular alkaloids from MeJA-elicitated cell strains C1 and C4, growing exclusively or in combination. The depicted extracted ion chromatograms are specific to different alkaloids; *m*/*z* 337 (catharanthine (CAT) and tabersonine (TAB)) and *m*/*z* 457 (vindoline (VDL)). **b** Simplified of the terpene-indole alkaloid pathway in *C. roseus*, and the differential partitioning of the branches in the two cell strains. R(C4) indicates a hypothetical regulatory factor from C4 cells that is able to advance the conversion of tabersonine into vindoline in C1 cells

Vincristine, a terpene indole alkaloid from Madagascar Periwinkle (*Catharanthus roseus*), belongs to the most potent and valued anti-cancer compounds, but accumulates only to minute amounts, because the complex pathway partitions to different cell types involving translocation into very few individual idioblasts sequestering vincristine. While a biotechnological alternative would be desirable, all attempts to produce vincristine in cell culture have failed so far (Shukla et al. 2010 and references therein). We wondered, whether this might arise from the fact that terpene indole alkaloid synthesis is subdivided into two concurrent (and, thus, competing) pathways (Fig. 7b). These mutually exclusive pathways proceed in different cell types in the *Catharanthus* leaf, which might be difficult to recapitulate in a biofermenter, where just one cell type prevails. To test this hypothesis, we generated cell strains from seedlings of *Catharanthus roseus* that differed in their metabolic potencies after elicitation by 100 methyl jasmonate (Fig. 7a). Strain C1 accumulated the precursor catharanthine and a higher level of tabersonine, while strain C4 accumulated a low level of tabersonine (while the catharanthine level was comparable to strain C1). None of the two strains accumulated any vindoline, the final step of the tabersonine pathway that, upon fusion with catharanthine, will generate the precursor of vinblastine and vincristine (Fig. 7b). However, a combination of C1 cells upstream of C4 cells in a circular set-up generated vindoline (Fig. 7a). At the same time, the tabersonine peak diminished strongly, consistent with a scenario, where tabersonine was converted to vindoline.

### Use of the chip to detect fungal phytotoxins in a plant-fungal ecosystem

Grapevine trunk diseases represent an emerging threat to viticulture. In contrast to classical pathogens, the responsible fungi can leaf as harmless for years but induce a so-called apoplectic breakdown of their host, when this host experiences severe stress, such as drought or heat (Khattab et al. 2021). There has been a debate, whether this breakdown results from the fungus clogging the vascular system of the host or from the secretion of phytotoxin. We used the chip system to simulate such a plant-fungal ecosystem and to probe for the potential secretion of such phytotoxins. To report bioactivity, we measured mortality by the Evans blue dye exclusion assay. In the absence of the fungus, the mortality of the tobacco BY-2 cells used as plant model was very low (around 10%). However, when we placed the same cells downstream of the fungal strain *Neofusicoccum parvum* Bt67, a known causative agent for the outbreak of the *Botryosphaeriacea*-related syndrome, a particularly severe form of grapevine trunk disease, mortality increased drastically to around 80%. This supports a model, where the fungus secretes a soluble phytotoxin and does not require direct contact with plant cells to induce their death.

## Discussion

Communication has been one of the main drivers of evolution. Communication is also one of the main drivers of development. In the very moment that multicellular life forms came into being, the differentiation between their cells required mutual signalling orchestrating the silencing of specific functions and the promotion of others. To ensure that the resulting organism remains functional, these hypo- and hypercellular functions must remain in balance (Lintilhac 1999). This is only possible through continuous and extensive exchange of information. To address intercellular signalling is methodologically challenging. While the cultivation of plant cells in vitro has been successful much earlier than for animal cells (Vasil 2008), a cell-biological approach to intercellular signalling has remained difficult, because plant cells in suspension have to be shaken. The current work originated from the motivation to develop a technical platform that would allow chemical interaction between plant cells while chaperoning against direct physical interaction. Our strategy was the development of a modular chip hosting plant cells under conditions that would retain their physiological status. In the following, we will briefly discuss the technical aspects of chip design and fabrication, before addressing the biological aspects of the three case studies, which we conducted to demonstrate possible applications.

We designed the fabrication of the microfluidic chip for short process times. For this, ultrasonic welding proved to be a rapid and suitable joining technique. By means of a two-step ultrasonic welding process, we sealed the cell chamber separately and enabled an in-plane integration of the tube fittings as fluidic interface for the chip. However, the fabrication could be optimised by using injection moulding to produce the housing parts. In contrast to hot embossing, the cycle times are significantly shorter and the fluidic connectors could be integrated directly. However, this requires more complex and therefore more expensive moulds.

The microfluidic chip is user friendly and compact for a quick experimental set-up and for observation by light microscopy. Since a sufficient supply of nutrients is crucial for a functional physiology, we simulated the mass transfer of the culture medium through the microfluidic chip and its membrane using sucrose as candidate molecule. The results of this simulation suggested that fluidic design and membrane porosity were suitable for perfusion cell experiments. In the next step, we used this chip for three case studies spanning a couple of potential applications.

In the first case study, we used a serial set-up of two chips loaded with tobacco BY-2 cells in different density to detect soluble factors that would restore proliferation in otherwise static cells (quorum-sensing factor). In fact, cells placed at a higher density in the donor chamber were able to significantly stimulate the proliferation of the cells in the recipient chamber (Fig. 6). The resulting proliferation factor (i.e. the ratio of cell density after 3 days of growth in the chip over the initial density of the inoculum) was in the range of that in flask cultures at comparable dilutions (Fig. 6a). Even the denser suspension (1:560 of a stationary culture) was still at the lower range of the dose–response curve, i.e. the proliferation factors can increase considerably, as shown by a previous study, where we had used BY-2 cells in similar chip in a flow-through set-up. Here, albeit at an around tenfold higher starting density, we observed a time course of mitotic index as in culture flasks (Maisch et al. 2016). Tobacco BY-2 cells are not the only model, where a quorum-sensing factor has become manifest. For instance, use of conditioned medium from densely growing cells was successful to induce growth of otherwise stationary *Taxus cuspidata* cells (Naill and Roberts 2005) or to rescue diluted carrot cell cultures from programmed cell death (McCabe et al. 1997). We are currently collecting the proteins secreted by densely growing cells to identify molecular candidates for this quorum-sensing factor.

Our second case study addressed modular synergy of different cell types differing in their metabolic potencies. The background for this experiment was a study on suspension cells from *Catharanthus roseus*, where we had succeeded to isolate strains differing in the activity of specific branches of the terpene-indole alkaloid pathway. The strain C1 accumulates the vindoline precursor tabersonine and, thus, recapitulates the metabolic competence of a leaf epidermal cell, while strain C4 accumulates only catharanthine and shows features characteristic for idioblasts (Liu et al. 2016). A serial array of two chips harbouring C1 cells produces as expected catharanthine and tabersonine, while a serial array of two chips harbouring C4 cells produces only catharanthine (Fig. 7a). None of these set-ups leads to detectable amounts of vindoline, a downstream product of the tabersonine branch of the pathway (Fig. 7b). This is not straightforward, since vindoline originates from joining the catharanthine moiety (present in C1) and a tabersonine educt (present in C1 as well). When we place a chip with C1 cells upstream of a chip with C4 cells, we obtain the desired vindoline (Fig. 7a), which represents a clear readout for metabolic synergy as it prevails in the real *Catharanthus* leaf. At the same time, the tabersonine peak is reduced, indicative of a conversion to downstream products. The bottleneck does not seem to be of metabolic nature, since strain C1 is accumulating similar amounts of catharanthine as C4 does. Our data rather suggest that a regulatory factor secreted by C4 cells (Fig. 7b, R(C4)) is able to overcome the bottleneck acting in C1 cells. Whether this regulatory factor activates the expression of metabolic genes in the later part of the tabersonine branch or whether it acts on compartmentalisation of catharanthine (that can exit cells through an exporter in the plasma membrane) is subject of ongoing research.

**Fig. 8:**
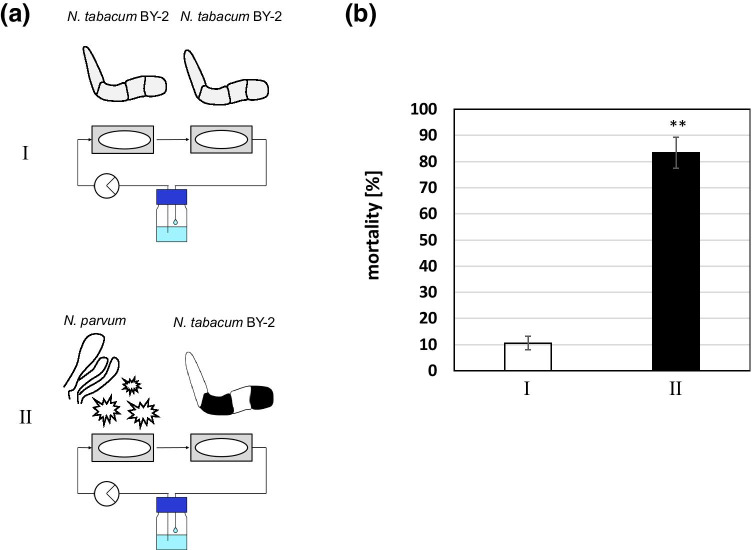
Demonstration of soluble phytotoxins secreted by the fungus *Neofusicoccum parvum* using tobacco BY-2 cells as readout system. **a** Design of the experiment. **b** Mortality observed under the two set-ups shown in (**a**). Data represent three biological replicates counting 550 to 4000 cells per replicate

In our third case study, we extended the system to the interaction between different organisms. Plant-pathogen interaction is of great relevance for agriculture. The spread of fungus-borne diseases destroys substantial parts of global harvest posing a threat for food security. How a fungus can attach and penetrate into the host cell has attracted considerable scientific attention. However, much earlier than the first physical contact, soluble signals decide the outcome of the encounter between both organisms. For instance, in mycorrhiza symbiosis, the host root can sense the approach of the symbiotic fungus before any physical contact (for review see Bonfante and Requena 2011). To separate physical penetration from chemical signalling is far from trivial, however. In the real tissue, both aspects occur in concert. By using a modular chip system, we could demonstrate that the phytotoxic effect of *N. parvum*, a pathogen that can cause apoplectic breakdown in grapevine (Botryosphariacea-related Dieback) does not require physical contact between fungal and host cells but depends on soluble signals secreted by the fungus (Fig. 8).

## Conclusions and outlook

In the current work, we have developed a modular chip system that allows mimicking technically the interaction of plant cells through chemical signalling. We have optimised design and fabrication for the use in plant cell biology and have exemplarily explored the use of this chip in applications of progressive complexity. Modular combination of cells of the same type (tobacco BY-2) enabled the detection of a soluble quorum-sensing factor. Modular combination of cells from different strains with different metabolic potency allows inducing the formation of the medically relevant alkaloid vindoline in *Catharanthus roseus*. Modular combination of cells of different life forms (the phytopathogen *Neofusicoccum parvum* and tobacco BY-2 cells) allows to detect the secretion of soluble phytotoxin and to exclude fungal penetration as cause for phytotoxicity. These applications demonstrate the potential of the modular chip system for analytical purposes. Modular arrays of higher complexity are conceivable, for instance, to study regulatory signals that result from a mutual sequence of signals, or that involve more than two partners. By parallelisation, the chip arrays can also serve to collect and identify the signals steering cell proliferation, metabolic competence, or defence against invaders. We currently extend the approach to additional systems, such as bacteria, or *Arabidopsis* seedlings, and also use the modular chip to test molecular candidates of the quorum-sensing signal.

## Data Availability

Data available on request from the authors.
